# Letter from Dr. Harlan

**Published:** 1839-03-02

**Authors:** 


### FOREIGN CORRESPONDENCE.

### LETTER FROM DR. HARLAN.—No. I.*

*Dinner at Professor Lawrence's—Visit to St. Bartholomew's Hospital—Visit to Bedlam—Visit to Guy's Hospital—Visit to Sir Astley Cooper—Visit to the College of Physicians—Professor Owen's Researches upon the intimate structure of the Teeth.*

LONDON, January 7th, 1839.

To the Editors of the Medical Examiner.

GENTLEMEN,—Agreeably to my promise to communicate to you any thing curious or new that might come under our notice during our professional visit to London and Paris, I now have the pleasure to address you a few lines, and place the letter at your disposal, supposing that there should be any thing in it worthy of public notice.

Three weeks have scarcely transpired since our arrival in London;—we have lost no time in our explorations, but we have seen much to admire and to gratify our professional curiosity. We had the honour to take our Christmas dinner with Professor Lawrence, 18 White Hall Place,

* No. 2 was published in No. 8 of the Examiner.

in company with some scientific friends. Mr. L. is already too advantageously known to most American physicians who have visited Europe, and partaken of his hospitality, to require any eulogiums from me. It is saying much to assert that his colloquial powers and talents to please equal those powers of composition which have already sent his name to all parts of the civilized world, and which will transmit it with honour to a distant posterity. The day following we met by appointment at St. Bartholomew's Hospital, and went the rounds with him. We learned that the cases of "Grande Verole" seldom presented themselves in such aggravated forms as with us: only two fatal cases occurred in one of the largest wards of this hospital during the last year. Professor L. pursues a medium path in the treatment of syphilis—administering mercury in some cases and stages. A convenient lecture room and dissecting rooms are connected with the hospital, whence we were inducted into the Anatomical Museum, rich beyond our most sanguine anticipations in pathological specimens, admirably prepared and preserved, as well as numerous preparations illustrative of human and comparative physiology and anatomy.

Professor L. prescribes at the hospital three times weekly, from 12 to 2, and lectures on surgery at 7 P. M., thrice weekly.

We next accompanied Mr. L. in his beautiful equipage to *Bedlam Hospital.* There, after being duly inducted into the mysteries of the Mènage, we were gratified with a view of several instructive cases of disease,—one, in particular, that of a maniac from epilepsy, who had attempted suicide by thrusting his head into burning coals,—the whole of the calvarium had exfoliated, and is preserved in the Museum of St. Bartholomew's. His disorder was in no manner relieved or affected by this severe cautery. The parts have been for many years covered with a hairless scalp. The brain is much flattened by atmospheric pressure, and its pulsations are very perceptible. Margaret Nicholson, who attempted the life of George III., and some of my old acquaintances among the crown prisoners, had paid the debt of nature,—Martin, who fired York Minster, in religious fanaticism, among the rest. Hatfield, who discharged a pistol at George III. in the theatre, still lives, and amuses himself with pelting small birds and quadrupeds, and writing doggerel verse. There are other prisoners condemned for life, being convicted of "crimes against nature."

Our attention was next directed to Guy's Hospital. This institution, formerly subsidiary to St. Thomas's, its opposite neighbour, is now second to no institution of the kind in Great Britain,—its museum, indeed, being unrivalled in the department of morbid anatomy. Nothing could be more complete and admirable than the representations in coloured wax of every genus, species, and variety of *cuticular* diseases, all scientifically arranged and ticketted,—thus rendering a class of disorders, exceedingly complicated and difficult to distinguish under ordinary resources, very easy of comprehension. Among the most striking of these, is the model of the upper half of the body of the patient who died a few months since, of "glanders," in its most exaggerated form—the history of which was detailed in the hospital reports, and attracted great attention from its rarity,—the eyes, nose, mouth, and all the mucous surfaces, appeared to be melting away, ulcerated, and discharging a copious sanies. Eruptions covered the fore part of the body, and gangrene had commenced about the shoulders and back. The sufferer had been a cab driver, and contracted the disorder by wiping the nose of his glandered horse with his handkerchief, and subsequently using it on his own person. Sir Astley Cooper first detected the nature of his complaint. It is somewhat remarkable that this noble and useful charity originated in the bad feelings of its founder, and has lately been further endowed by a similar cause. Mr. Guy, it appears, was a miserly bookseller, who amassed a large fortune by trade, and increased it by his frugality. He took a notion to marry his maid or housekeeper in his old age, and dissolved the engagement in a rage, when he found, on his return one day, that his intended had exceeded his commands in some repairs he had ordered to be made in the pavement before his door, in preparation for his marriage. The lady, calculating too surely on her conquest, instructed the workmen to add a few more bricks, on her own responsibility,—on discovering which, Mr. G. drove her from his house, and willed all his property to endow the hospital which bears his name. The recent accession of half a million dollars was obtained from a retired merchant of rather aristocratical notions. He had intended to leave his fortune to a brother, who, on dining with him one day, unceremoniously took a potato in his fingers and dipped it into the saltcellar. On being reproved for his indecency, he answered, that his brother and his potato might both be d——d! He was, in consequence, disinherited, and Guy's Hospital endowed. There have been added since my visit in 1833, a new cabinet for the museum, and other valuable additions have been and are daily making. B. Cooper, Bright, &c., are now prescribing and lecturing here. These surgeons have able emulators in Travers, Tyrrel, Green, &c., at St. Thomas's, which was formerly the theatre of Sir Astley Cooper's operations, and is a more ancient institution than Guy's, having been founded by Edward VI., who died in his youth, and whose bronze statue in the square of the hospital is one of the most graceful and perfect specimens of art. We were kindly inducted here by Mr. Solly, who lectures on comparative anatomy and physiology in this institution. He directed our attention to some fine specimens of injected morbid preparations,—many by the hands of Sir Astley himself. The opportunities for pursuing practical anatomy appeared to be on rather a larger scale, than in most other places in London; but even this will bear no comparison with similar facilities in Paris, or even in the United States. On inquiring the present price of subjects, we were informed that students did not think of appropriating a whole subject to themselves, but paid about two dollars each for the eighth of a subject, and that in some institutions they could obtain none during the whole winter!

At one time, twenty or twenty-five guineas were demanded by the "jackalls" for each subject;—this was during the excitement created by the developements made on the trial and conviction of Hare and Burk—the excitement and terror of the citizens being carried so far, as to induce them to remove the bodies from whole grave-yards. A teacher of practical anatomy, (Mr. Miller, I think,) who endeavoured to oppose the monopoly and extortion of the "dealers," and obtained subjects necessary for his demonstrations by his own personal labour, was informed against by the "trade,"—was convicted, and died in jail!

It is the custom, at this season of the year, for the "haut-ton" to leave the city, and enjoy the Christmas festivities at their country seats,—we thus twice failed in our attempts at a personal interview with Sir Astley Cooper, but we were richly repaid for our trouble when we at last succeeded in seeing recently the cynosure of British surgery, now in the seventy-first year of his age, and the fifty-fifth year of his career. He appears but little altered during the five years elapsed since my last interview. Replete with honours and riches, he enjoys the blessings of a green old age—his intellect still unshattered. He revels in past recollections, and he discourses on subjects connected with his profession with the enthusiasm of youth. Up to this moment, he amuses himself and instructs the world by his physiological investigations and experiments on living animals. We were delighted with the clear and admirable manner in which he illustrated some of the most important functions of the human body. There was much novelty to us in his mode of explaining his views of the "secondary nature of the nervous system." His motto would appear to be, "Omnia ex sanguine." He has proved by experiment that the carotid arteries have little, if any, connexion with the functions of the brain, these depending principally on the *vertebral arteries*. He showed us admirable preparations, made by his own hands, of the dog and rabbit, in which the carotids in some cases, and the vertebrals in others, and in some instances where all four of these important vessels had been tied, and where the anastomosis continuing the circulation constituted a beautiful "rete mirabile."

His memoirs on this interesting subject are published in Guy's Hospital Reports, No. 3.

Sir A. kindly presented us with copies separately published, and illustrated by finely coloured plates. Numerous other specimens of anatomy, prepared by his own hands, were brought forward, and lucidly explained,—indeed, many of his minute injections, both with mercury and the usual fine injection materials, for the illustration of some of the more obscure and important organs of the body, are acknowledged by all who see them to surpass every thing of the kind.

Numerous engagements for the day obliged us to withdraw from the society of this distinguished surgeon and admirable man, much sooner than was desirable on our part; not, however, before he had expressed a just tribute to the memory and talents of our beloved Physick—inquiring after Dr. Warren—offering me in the kindest manner letters to distinguished surgeons in Paris—giving his card as an introduction to all the hospital surgeons of London, and inviting us to return on an appointed day, to witness some physiological experiments on a living rabbit, an opportunity we have no disposition to neglect.

We next proceeded to visit the Hall of the *College of Physicians*, a magnificent new building in Pall Mall East, erected in 1825. The

"College" was founded in 1518 by Dr. Linacre; an act of incorporation was obtained from Henry VIII. As a professional body, the college has slept of late years—though formerly possessed of great powers and privileges. Recently they have shown some manifestations of a determination to arouse themselves from their lethargy, and have issued a proclamation declaring their intention of resuming the power invested in themselves alone to grant degrees or license to practice physic—a privilege they have permitted occasionally to pass into the hands of other lecturers. It is their intention to confer the degree upon all who submit to and pass examination, independently of the when and where and with whom the candidate may have studied medicine. This medical "ukase" of the college has not been well received by the practitioners of London.

The basement story of the "College" is occupied by small rooms for offices on one side, and by a large dining room, the entire length of the building, on the other side—containing a large oaken table in the middle, and wainscoating of the same material, all brought from the old Hall. Hung round are good portraits of Drs. Meade, Garth, Heberden, Hale, the two Tysons, &c. The library and museum principally attracted our regard, the latter being the depository of the celebrated preparations by which the immortal Harvey illustrated the circulation of the blood. They consist chiefly of the *arteries* of the whole human body carefully dissected out, so as to preserve their continuity, spread whilst recent on a smooth board and dried, as in some of the old Roman preparations—there are two others, also of adult size, with the veins and nerves similarly prepared—these were deposited in 1654, and are in pretty good preservation, with the exception of some parts of the smaller veins, which sacrilegeous hands have barbarously abstracted as relics. Further depredations have been obviated by coarse brass nettings or frames, at present placed before these sacred monuments of the imperishable renown of the author. The museum contains other rare and curious anatomical pieces well worth examination, both from their intrinsic value, and from the frequent references made to them by able and respected authorities. The library is extensive, but appeared rather antiquated—portraits of Harvey and Ratcliff embellish this room. A censor's room and lecture room are also on the second floor—they contain a fine bust of Sydenham, and another by Chantrey of Sir H. Halford—an admirable half length portrait of Vesalius from the Italian school—of Sir Hans Sloan, Glisson, Baily, Meade, Sydenham, Heberden, the two Hunters, and numerous other eminent physicians—also, an interesting picture representing John Hunter lecturing on anatomical proportions from a living man, before the school of design, &c. &c.

We have not time at present to detail observations made during frequent visits to the Royal College of Surgeons, or our interview with Dr. James Johnson, and other eminent physicians. We have accepted invitations to dine with the clubs of the Royal Society—Linnean, Geological, and Zoological Societies—all of which will afford ample resources for future communications.

We must now proceed to mention the results of an evening delightfully passed with Mr. R. Owen, curator of the museum, in examining a novel subject of anatomical research.

*December 31st.*—In company with Mr. Van Buren, we visited, by invitation, Mr. Richard Owen, curator of the Royal College of Surgeons, at 6½ P. M., for the purpose of examining with the microscope numerous specimens of the teeth of various animals, recent and fossil, prepared by Mr. O. with the view of illustrating the intimate structure of these organs, in a memoir which he is now preparing for the Philosophical Transactions, London.

According to Mr. O.'s investigations, the human tooth consists of enamel, ivory, and the "*pars petrosa*," or cement; or, of a central pillar of ivory, covered by enamel on the crown and body, and with cement around the root and cervix. The structure of the ivory is essentially tubular, consisting of animal cylinders or tubes proceeding from the central cavity transversely to the axis of the tooth, to the sides, and perpendicularly to the crown, so as to oppose their distal extremities to the points of greatest pressure: these tubes he names "calcigerous," as they are found to contain the earthy part of the tooth; they are occasionally observed to extend beyond the line of juncture of the ivory with the enamel, as if for the purpose of increasing the attachment between the two surfaces, but are generally received, and are lost in the body of the ivory; each of these tubes is separated from its neighbour by a space greater than its own diameter, which space is filled by lateral tubes of a similar nature, and terminating in cells. The tubular structure of this ivory, Mr. O. is well aware, was not only known, but adequately illustrated, by

*Lewenhœck.* These calcigerous tubes do not run in a straight line, but their general tendency is a curvature upwards, and, in addition to which, each line or tube is distinctly waved in itself. These facts Mr. O. lucidly illustrated in numerous prepared specimens from the teeth of the human subject, those of the shark, both fossil and recent, the horse, the fossil icthyosaurus, iguanodan, the elephant, the megatherium, dinotherium, several fishes fossil and recent, and others equally instructive.

Mr. O. further illustrated his subject, by referring to the pathology of these organs; he had examined numerous decayed teeth, in every instance of which the caries had commenced in the space or position beneath the enamel, between it and the ivory, and had extended itself towards the central cavity in the direction of the calcigerous tubes, a phenomenon by no means explicable by the usually received notions of the laminated structure of the teeth. The original cause of decay in these cases, Mr. O. refers to the generation of an acid, or at least, of a fluid which soon becomes acid, between the enamel and the ivory.

Mr. O. next proceeded to illustrate the structure of enamel, by means of the same excellent microscope of Pritchard; this striated structure is of a crystalline texture, the striæ consisting of hexahedral prisms, always arranged at right angles with the superfices, a disposition which offers the greatest resistance to friction; these striæ differ in color and density from the ivory.

The "pars petrosa," or cement is always *tubular* in structure, appearing under the microscope as lines interspered with minute black dots, or corpuscles; these tubes he denominates "*medulliferous*," inasmuch as they carry marrow; and in most other respects, this cement resembles ordinary bone in structure and composition. Another important discovery due to the researches of Mr. O. is the peculiar structure of the teeth of the megatherium; these were formerly described by Cuvier as pillars of bone surrounded and traversed by enamel. Mr. O. demonstrates that portion of these teeth usually considered as enamel, to consist of a finer and denser kind of ivory; the body of these teeth consists, therefore, of common ivory, traversed by transverse ridges of denser ivory, and surrounded by cement, or pars petrosa. In all cases these interesting and novel facts were abundantly illustrated by numerous objects, both of human and comparative anatomy, and bore ample testimony to the acumen and laborious research of the experimenter, and of the important results to physiology to be derived from investigations of comparative anatomy, and which reflect on our neglect at home of this indispensable department of experimenal philosophy. You will find a notice of these admirable investigations of Mr. Owen, in the account of the last meeting of the "British Association" at Newcastle published in the London Athenæum, which will be completed "in extenso" in the forthcoming volumes of the "Transactions of the Royal Society." The "Memoirs" will appear in Decades, and be beautifully illustrated.

We were further impressed by a most curious fact connected with these interesting microscopic investigations of Mr. O. viz: that the different *species* of animals were shown to be recognisable by specific arrangements of their dental structure!

